# Supplementary material for: Multivalley Free Energy Landscape and the Origin of Stripe and Quasi-Stripe CDW Structures in Monolayer MX2 Compounds
Source: Sci Rep. 2020 Jan 27;10:1239. doi: 10.1038/s41598-020-58013-7 (PMC6985243; doi:10.1038/s41598-020-58013-7)
Supplement: Supplementary file 1 — Supplementary Information. [file 41598_2020_58013_MOESM1_ESM.docx]

**Supplementary Information: “Multivalley Free Energy Landscape: Origin of Stripe and Quasi-Stripe CDW Structures in Monolayer MX_2_ compounds”**

Keiji Nakatsugawa^1,2^^[[1]](#footnote-1)^^[[2]](#footnote-2)^, Satoshi Tanda^1,2*^^[[3]](#footnote-3)^ and Tatsuhiko N. Ikeda­^3*^^[[4]](#footnote-4)^

1*Department of Applied Physics, Hokkaido University, Kita 13, Nishi 8, Kita-ku, Sapporo, 0608628, Hokkaido, Japan. Tel.: +81-11-706-6154, Fax: +81-11-706-6154*

2*Center of Education and Research for Topological Science and Technology, Hokkaido University,*

*Kita 13, Nishi 8, Kita-ku, Sapporo, 060-8628, Hokkaido, Japan. Tel.: +81-11-706-6154, Fax: +81-11-706-6154*

3*Institute for Solid State Physics, University of Tokyo, Kashiwa, 277-8581, Chiba, Japan*

The intralayer mechanism of the incommensurate-striped phase boundary was first studied by Littlewood and Rice [1] where the stripe free energy minimum can be a global one due to competition between the elastic and commensurability energies. So, we study the dependence on the coefficient of commensurability energy to investigate the consistency of our result.

According to Littlewood and Rice, the difference between $F_{\mathrm{Incomme}}$ (free energy for the hexagonal incommensurate phase) and $F_{\mathrm{Stripe}}$ (free energy for the stripe phase) is given by

$$F_{\mathrm{Stripe}}-F_{\mathrm{Incomme}}=\frac{1}{2}q^{2}+0.5\psi_{2}-e\psi$$

(see Equations (13) and (14) in ref. [1] and neglect the interlayer coupling, i.e., $f=0$). Here, the $q^{2}/2$term corresponds to elastic energy, $\psi_{2}\sim0.1$ (inferred from Fig. 2 in ref. [1]) is a small contribution from the second harmonic, and $e\psi$ is the commensurability energy. In the Nakanishi-Shiba model [2], $\frac{Y}{2}=-b_{1}/8$ corresponds to the parameter $e$.

Littlewood and Rice used a positive value of $e$, so $F_{\mathrm{Stripe}}-F_{\mathrm{Incomme}}$ can be negative and $F_{\mathrm{Stripe}}$ for the stripe phase can be a global minimum. On the other hand, we use a negative value $Y=-0.3$ following ref. [2]. In Figure S1 we have calculated the general Y-dependence of the free-energy difference between the stripe and incommensurate phases. We see that the stripe (incommensurate) phase is more stable for the positive (negative) Y. This is why the stripe phase is a local minimum in our calculation while it is a global minimum in [1].

Figure S1: General Y-dependence of the free-energy difference between the stripe and incommensurate phases.

| [1] | P. B. Littlewood and T. M. Rice, "Theory of the Splitting of Discommensurations in the Charge-Density-Wave State of 2H-TaSe2," *Phys. Rev. Lett.,* vol. 48, no. 1, p. 27, 1982. |
| --- | --- |
| [2] | K. Nakanishi and H. Shiba, "Domain-like Incommensurate Charge-Density-Wave States and the First-Order Incommensurate-Commensurate Transitions in Layered Tantalum Dichalcogenides. II. 2H-Polytype," *Journal of the Physical Society of Japan,* vol. 44, p. 1465–1473, 1978. |

1. Corresponding author. [↑](#footnote-ref-1)
2. E-mail: keiji_nakatsugawa@eis.hokudai.ac.jp [↑](#footnote-ref-2)
3. E-mail: tanda@eng.hokudai.ac.jp [↑](#footnote-ref-3)
4. E-mail: tikeda@issp.u-tokyo.ac.jp [↑](#footnote-ref-4)
